# Effect of miR-146 targeted HDMCP up-regulation in the pathogenesis of nonalcoholic steatohepatitis

**DOI:** 10.1371/journal.pone.0174218

**Published:** 2017-03-27

**Authors:** Xi Jin, Jiang Liu, Yi-peng Chen, Zun Xiang, Jie-xia Ding, You-ming Li

**Affiliations:** 1 Department of Gastroenterology, The First Affiliated Hospital, College of Medicine, Zhejiang University, Hangzhou, China; 2 Department of Gastroenterology, Huzhou Central Hospital, Huzhou, China; 3 Department of infectious disease, Hangzhou first people's hospital, Hangzhou, China; University of Basque Country, SPAIN

## Abstract

**Backgrounds/Aims:**

Mitochondrial dysfunction plays an important role inthe pathogenesis of nonalcoholic steatohepatitis (NASH), where uncoupling protein (UCP) is actively involved. We previously reported the uncoupling activity of HDMCP and its role in liver steatosis. We now aim to investigate the degree and therapeutic effect of HDMCP in NASH and the regulatory role of miR-146 on HDMCP.

**Methods:**

NASH animal model was established by feeding BALB/c mice with MCD diet while L02 cell was cultured with high concentration of fatty acid (HFFA) for 72h to mimic the steatosis and inflammation of NASH in-vitro appearance. The steatosis level was assessed by H-E/oil-red staining and serum/supernatant marker detection. The inflammation activity was evaluated by levels of Hepatic activity index, transwell, apoptosis degree (TUNEL/flow cytometry) and serum/supernatant marker. HDMCP level was detected by western blot and miRNA expression was tested by qRT-PCR. NASH severity change was recorded after RNA interference while the regulatory role of miR-146 on HDMCP was confirmed by dual luciferase report system. The H_2_O_2_ and ATP levels were measured for mechanism exploration.

**Results:**

Increased HDMCP expression was identified in NASH animal model and HFFA-72h cultured L02 cell. Moreover, under regulation of miR-146, NASH alleviation was achieved after HDMCP downregulation in both in vivo and in vitro, according to the declination of steatosis and inflammation related markers. Though H_2_O_2_ and ATP levels were increased and decreased in NASH models, HDMCP down regulation both increased their levels.

**Conclusions:**

The miR-146-HDMCP-ATP/H_2_O_2_ pathway may provide novel mechanism and treatment option for NASH.

## Introduction

Nonalcoholic fatty liver disease (NAFLD) is defined as a common clinicopathologic condition characterized by lipid deposition in hepatocytes, precluding excessive alcohol intake[1]. The incidence of NAFLD has gradually increased, reaching approximately 20% in the world[2] and 15% in China[3]. Nonalcoholic steatohepatitis (NASH) is an important stage in NAFLD for its characteristic of inflammation initiation and end-stage liver disease progression. It has been regarded as a significance cause of cryptogenic cirrhosis[4] and liver transplantation[5]. Currently, the pathogenesis of NASH is still vague, where accumulating evidences supported the vital role of mitochondrial dysfunction [6]. Theoretically, hepatic mitochondria are the major site of fatty acid metabolism and the concomitant oxidative stress may accelerate the transition from simple steatosis to NASH. However, though mitochondrial morphology change such as structure damage[7] and permeability transition pore opening[8] have been reported, the functional change and specific protein mediated mitochondrial dysfunction were rarely investigated.

Uncoupling proteins (UCPs), member of the mitochondrial anion-carrier protein superfamily, uncouple mitochondrial respiration from ATP synthesis by dissipating the transmembrane proton gradient to further affect mitochondrial function and metabolic processes[9]. It is well acknowledged that mitochondrial proton leak accounts for 20–30% of the oxygen consumption of isolated resting hepatocytes[10], indicating the existence of protein exerting uncoupling activity and UCPs are suitable candidates. However, none of UCPs (UCP1-5) were detected in normal hepatocytes. Moreover, whether UCPs are expressed in NAFLD is still in contradictory. For instance, Baffy G et al reported that obesity related fatty liver is unchanged in mice with UCP2 knockout [11]. However, a relatively new study supported the increased UCP2 level in NASH[12]. Those discrepancies make it become necessary and urgent to find novel UCPs and explore their effects in NASH.

Hepatocellular carcinoma down regulated mitochondrial carrier protein (HDMCP) was first cloned and reported to bear all the hallmark features of the mitochondrial anion-carrier proteins in the year 2004 [13]. We further confirmed its uncoupling activity in a yeast expression system and first showed the increased HDMCP level in steatosis stage of NAFLD, where the involvement of decreased ATP and H_2_O_2_ production was identified as underlining mechanism[14]. Nevertheless, the effect of HDMCP and its regulator and effector in NASH is still unclear. Furthermore, aberrantly expressed miRNAs were reported in different stages of NAFLD, highly supporting the possibility of the existence of certain miRNA in regulating HDMCP expression. Therefore, we investigated the expression level and therapeutic effect of miR-146-HDMCP-ATP/H_2_O_2_ pathway in NASH model, aiming to provide novel concept for NASH pathogenesis and therapy.

## Materials and methods

### Ethic statement

This study was carried out in accordance with the recommendations in the Guide for the Care and Use of Laboratory Animals of the National Institutes of Health. The protocol on animal was approved by the institutional review board of the First Affiliated Hospital of Zhejiang University.

### The pathology and serology markers of NASH animal model

A total of 16 male BALB/c mice aged 6 week were purchased from Cavens Lab Animal (Suzhou, china) and randomly divided into two groups: NASH (n = 8) and control (n = 8). All mice received food and water ad libitum and were maintained on a 12/12-h light/dark cycle. Control group was given a basic diet while NASH group was given a MCD diet for 4 weeks as previously reported [15]. Mice were sacrificed by neck dislocation at appointed time spot, where blood and liver tissue was collected for further analysis. After body weight detection, liver sections were stained with Haematoxylin-Eosin (H-E) and observed for hepatic steatosis and inflammation by Olympus microscope. Besides, histological activation index (HAI) was calculated to semi-quantitatively evaluated the severity of hepatic injury as previously reported[16].

Firstly, serum triglyceride (TG)and supernatant Cholesterol (Tch) were tested with Hitachi 7600 clinical analyser (Department of laboratory, the First Affiliated Hospital of Zhejiang province). Secondly, alanine aminotransferase (ALT) and aspartate aminotransferase (AST) that were leaked from injured hepatocytes were considered as the indirect markers of liver inflammation and tested using the same method. Thirdly, tumor necrosis factor-α(TNF-α), interleukin-1β (IL-1β), interleukin-6(IL-6) and interleukin-18(IL-18) were detected using ELISA methods (BOSTER Biotechnology Limited, Wuhan, China). Fourthly, Malondialdehyde (MDA) level as the indirect reflector of reactive stress was tested by routine TBA method (Nanjing Jiancheng Bioengineering Institute, Nanjing, China). H_2_O_2_ level was assessed based on its ability in binding molybdenic acid to form a complex using a commercially available kit (Nanjing Jiancheng Bioengineering Institute, China). Finally, mitochondrial ATP level was tested as previously reported [14].

### Steatosis and inflammation induction in L02 cell treated with high concentration of free fatty acid (HFFA)

L02 cell was purchased from China Cell Culture Center (Shanghai, China) and routinely cultured under condition of 5% CO_2_/95% air at 37℃. Cell viability was tested by trypan blue exclusion before any experiment and the viability over 90% was considered eligible. Those eligible L02 cells at 80% confluency were further exposed to HFFA, a mixture of oleate (OA) and palmitate (PA), at the final ratio of 2:1 and final concentration of 1 mM for 48 and 72h, respectively. L02 cells were divided into control, HFFA-48h and HFFA-72h groups and then harvested for detection of steatosis and inflammation.

For lipid droplet observation, harvested L02 cells were rinsed with PBS and fixed with 10% neutral formalin for 30 min. They were then dyed with Oil red (1 mg/ml in PBS) at 37℃ for 20 min as Oil red could easily label fat accumulation in the cytosol based on its lipophilic characteristics. After rinsed with PBS again, L02 cells were observed by microscope. Other steatosis, inflammation and oxidative related markers detection from L02 cell supernatant were the same as those in NASH animal model.

### Apoptosis analysis and transwell test

Apoptosis in HFFA cultured L02 cell was analyzed using Annexin V-EGFP/PIdouble dying method by flow cytometry according to the manufacturer’s instructions (Shanghai R&S Biotechnology Co., Ltd, China). Cells from HFFA-72h and control groups were digested by trypsin, centrifuged at 1000rpm for 5 min, washed by PBS for two times and re-suspended in 400ul binding buffer. Thereafter, 5ul Annexin V-FITC and 10ul PI were consecutively added and incubated in black for 30min at 20℃. Thereafter, apoptosis was assessed by dual-color flow cytometry on a FACScan cytofluorometer (BD Bioscience) using CellQuest software (BD Bioscience).Additionally, apoptosis in NASH animal model was accomplished by TUNEL method (100 Biotech, Hangzhou, China) as previously reported [17], where apoptosis index = (apoptotic cell/total cell)*100%.

Crystal violet staining (GenMed, Minnesota, the USA) in a co-culture system of transwell plates to test the inflammatory cell infiltration ability of HFFA-cultured L02 cells was applied, based on its ability of being absorbed by cells colonized in monolayer[18]. Transwell method was applied as previously described while HL-60 cell was chosen to represent inflammatory cell in NASH for its neutrophil like phenotype[19]. In detail, 0.1ml vital HL-60 cell (1×10^5^/ml) was added into a small cabin (Corning, NY, the USA) and then each cabin was imbedded into a 24 plate that was filled with pretreated L02 cells from control and HFFA-72h groups. These two types of cells were co-cultured for 48h and then supernatants from 24 plates were collected. After centrifugation, the sediments were further fixed with 4% formaldehyde and dyed with crystal violet. The dyed cells were observed under microscope and then lysed. Crystal violet released from those lysed cells was further measured by its absorbance value under 570nm.

### In vivo and In vitro RNA interference

Firstly, the miRNA oligos designed to bind different sites of HDMCP were synthesized and linked to a well acknowledged pcDNA^TM^6.2-GW/EmGFP-miR expression plasmid (Invitrogen, the USA, Fig A in S1 File). After E. Coli (DH5α) transfection and incubation, those 4 plasmids were extracted and the correctness of their sequence was verified by gene sequencing. Secondly, 293T cell was transfected with POLO3000 to test the ability of these four plasmids in deceasing HDMCP expression, where GAPDH was used as internal control. Plasmid 3 was selected for further interference study as its efficacy in decreasing HDMCP expression reached as high as 57% (Fig B in S1 File). Finally, L02 cell was transfected with different regents using POLO3000 and was divided into four groups: control, NASH, NASH+HDMCP miRNA and NASH+NC miRNAfor further study.

Previously synthesized miRNA oligos were also used as in-vivo siRNAs after cholesterol modification and HPLC purification. In detail, mixture of four siRNAs (50 ug regents in 1.0ml PBS) was injected into caudal vein of mice model with the frequency of twice every week. In this step of experiment, 32 BALB/c mice were divided into four groups receiving different regents as followings: control+ NC-mouse-siRNA oligos, control+HDMCP-mouse-siRNAoligos, NASH+NC-mouse- siRNA oligos and NASH+HDMCP-mouse-siRNA oligos. After 4 weeks feeding, mice were sacrificed at 24h after last caudal vein injection. Their liver tissue and blood were collected for further analysis.

### Regulation of miR-146 on HDMCP level

The regulatory role of miR-146 on HDMCP was tested with dual luciferase report system. Firstly, the normal gene fragment containing 3’ UTR region of HDMCP wide type and the specific dual-luciferase miRNA target expression vector-pmirGLO (Promega, the USA) were both cut with SacⅠand SalⅠ. These two segments were then connected with T4 DNA ligase (Fermentas, Lithuania) at 22℃ for 2h. Secondly, the combined vector pmirGLO-HDMCP-3’UTR was used to transfect competent cell with CaCl_2_. Thereafter, the transfected cells were cultured at 37℃for 16h, followed by pmirGLO-HDMCP-3’UTR extraction (TIANGEN, China) and verification by gene sequencing. Thirdly, the miR-146 mimics were synthesized and cotransfected both BRL-3A and L02 cells with pmirGLO-HDMCP-3’UTR using lipofectamin 2000 (Invitrogen, The USA). After culturing at 37℃/5% CO_2_ for 25h, those cotransfected cells were harvested and the luciferase activity was tested using a dual-luciferase reporter gene detection kit (Promega, the USA).In this step, subjects were divided into four groups as followings: blank cell group, pmirGLO-HDMCP-3’UTR group, negative control miRNA+pmirGLO-HDMCP-3’UTR group and miR-146+pmirGLO- HDMCP-3’UTR group.

### Quantitative real-time PCR and western blot

For miRNA quantitative analysis in HFFA cultured BRL-3A cells, 2μg of retrieved total RNA was reversely transcribed using stem-loop antisense primer mix and AMV transcriptase (TaKaRa, China). Real-time PCR was routinely performed on MX3000p real time PCR system (Stratagene, USA). U6 snRNA was amplified as a normalization control and the relative amount of each miRNA to U6 RNA was calculated using the equation 2^-∧CT^, where∧CT = C_TmiRNA_-C_Tu6_. HDMCP protein level was quantified by routine western blot with the primary mouse polyclonal antibody raised against HDMCP (SANTA CRUZ, sc-161699) and an ECL chemiluminescence kit (Santa Cruz, USA) in NASH mice models and L02 cells cultured with HFFA for 48h and 72h. The normalization was performed by blotting the same samples with a mouse anti-GAPDH antibody.

### Bioinformatics analysis, statistics

Statistical analyses were performed using SPSS, version 16 (SPSS, Chicago, IL, USA). Data are presented as the mean ± standard deviation when data were found to be normally distributed or as the median if the distribution was skewed. Differences between groups were analyzed using the Student’s *t*-test or the Mann–Whitney *U* test.

## Results

### Increased HDMCP expression in successfully established NASH animal model

**Fig 1:**
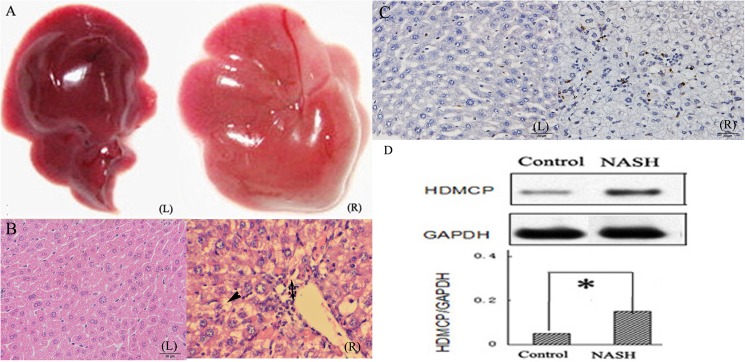
Increased HDMCP expression in NASH mice model. (A), general image of liver tissue; (B), H-E staining of liver tissue (Single arrow indicates lipid droplet; double arrow indicates inflammatory cell infiltration); (C), apoptosis degree (shown in Table 1) was calculated on the basis of the percentage of apoptotic cell that was shown in brown color, while normal cell was in blue-purple color by Tunel method. (D), relative hepatic HDMCP expression in NASH (n = 8) and control group (n = 8). (L) indicates control group; (R) indicates NASH group; *, p<0.05.

NASH mouse model was successfully established after feeding MCD diet for 4 weeks, as reflected by distinctive changes in both serum and liver tissue. Generally, mice from NASH group showed yellow-enlarged liver as well as hepatic fat deposition, hepatocellular ballooning, mild to moderate chronic portal and intra-acinar inflammation (Fig 1A & 1B), as confirmed by an independent pathologist. Moreover, inflammation (hepatic HAI, serum ALT, AST, TNF-α, IL-1β, IL-6, IL-18, apoptosis degree) and oxidative stress related (serum MDA, mitochondrial H_2_O_2_) biomarkers were significantly increased while body weight, TG and mitochondrial ATP level was significantly decreased in NASH group than those in controls (Fig 1C, Table 1). Finally, HDMCP level was significantly increased in NASH group as shown by western blot (Fig 1D).

**Table 1:** Changes in hepatic and serologic markers in NASH animal model.

	Control	NASH	p
body weight (g)	22.52±3.21	13.31±2.76	<0.01
HAI	0.75±0.18	2.97±0.46	<0.01
TG(mmol/L)	2.50±0.28	0.78±0.16	<0.01
ALT(mmol/L)	49.97±4.06	82.42±10.29	<0.01
AST(mmol/L)	54.79±5.48	110.24±10.78	<0.01
TNF-α(ng/L)	0.71±0.16	1.46±0.25	<0.01
IL-1β(ng/L)	5.52±0.61	12.89±1.38	<0.01
IL-6(ng/L)	57.46±4.86	113.42±10.18	<0.01
IL-18(ng/L)	9.40±0.75	22.50±2.79	<0.01
apoptosis degree (%)	2.40±0.82	7.31±0.82	<0.01
MDA(nmol/L)	8.83±0.87	13.72±1.34	<0.01
H2O2(mmol/g protein)	22.61±3.84	56.93±5.10	<0.01
ATP(mmol/g protein)	5.52±0.70	3.28±0.44	<0.01

HAI, histological activation index; TG, triglyceride; ALT, alanine aminotransferase; AST, aspartate aminotransferase; TNF, tumor necrosis factor; IL, interleukin; MDA, Malondialdehyde; ATP, adenosine triphosphate. HAI, apoptosis degree, H_2_O_2_and ATP were measured in liver and the rest of the parameters were measured in serum.

### Increased HDMCP level in HFFA cultured L02 cell with steatosis and inflammation formation

**Fig 2:**
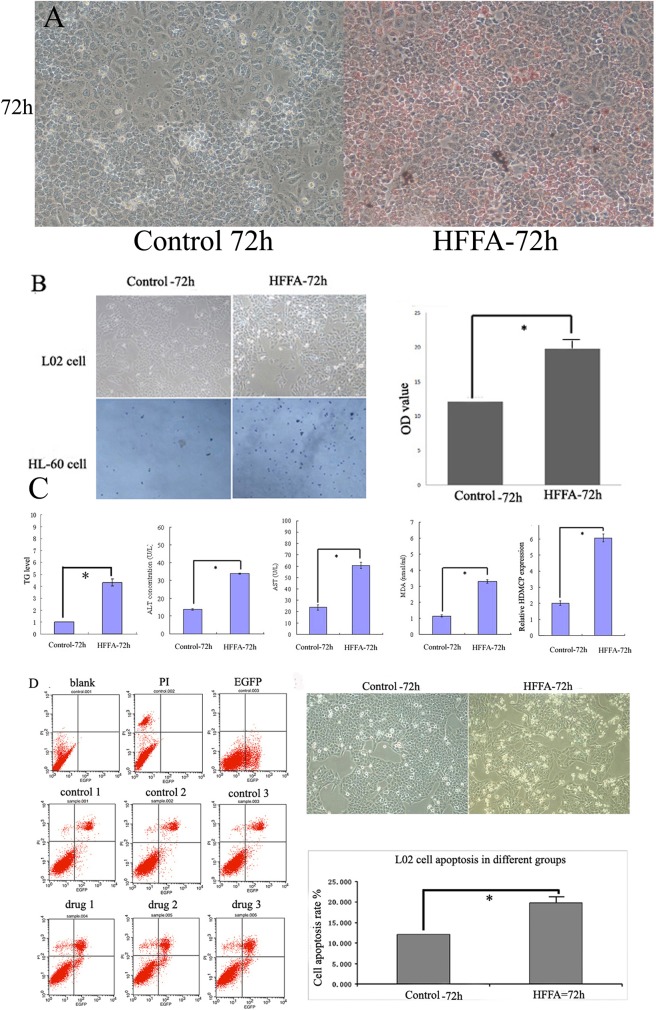
Increased HDMCP level in HFFA-72h group. (A), oil red staining showed increased lipid accumulation (red droplet) in HFFA-72h group. (B), transwell experiment showed increased inflammatory cell infiltration in HFFA-72h group as more HL-60 cells were migrated from the cabin to the 24-well plate in HFFA-72h group. The increased OD value from right panelshowed more crystal violet staining HL-60 cells in HFFA-72h group. (C), significantly increased TG, ALT, AST, MDA and HDMCP levels in HFFA-72h group. (D), increased apoptosis in HFFA-72h group. Left panel showed theimage of L02 flow cytometry.Right upper panel showedthe microscopic image of increased round and shrunk L02 cells in HFFA-72h group. Right lower panel showed the significantly increased L02 cell apoptosis in HFFA-72h group based on repetitive L02 flow cytometry. *, p<0.05. All cell experiments were carried out with three repetition.

After culturing with HFFA for 72h, L02 cell was harvested, stained and fully analyzed. Firstly, the oil red stained lipid droplet was significantly increased in HFFA-72h group, indicating the formation of steatosis (Fig 2A). Secondly, transwell method was used to assess the ability of inflammatory cell infiltration that is an important character of NASH pathology. As shown in Fig 2B, the amount of HL-60 cells was significantly higher in the 24-well plate in HFFA-72h group. Moreover, after staining with crystal violet, the OD value in HFFA-72h group was also significantly higher, indirectly indicating the increased inflammatory cell migration. Thirdly, the supernatant TG, ALT, AST, MDA and cellular HDMCP levels were significantly increased in HFFA-72h group (Fig 2C), further supporting the appearance of hepatocyte steatosis and injury. Finally, the increased L02 cell apoptosis was also presented in HFFA-72h group through flow cytometry (Fig 2D).

### HDMCP mediated in vivo and in vitro NASH alleviation through ATP depletion and inflammation activation

**Fig 3:**
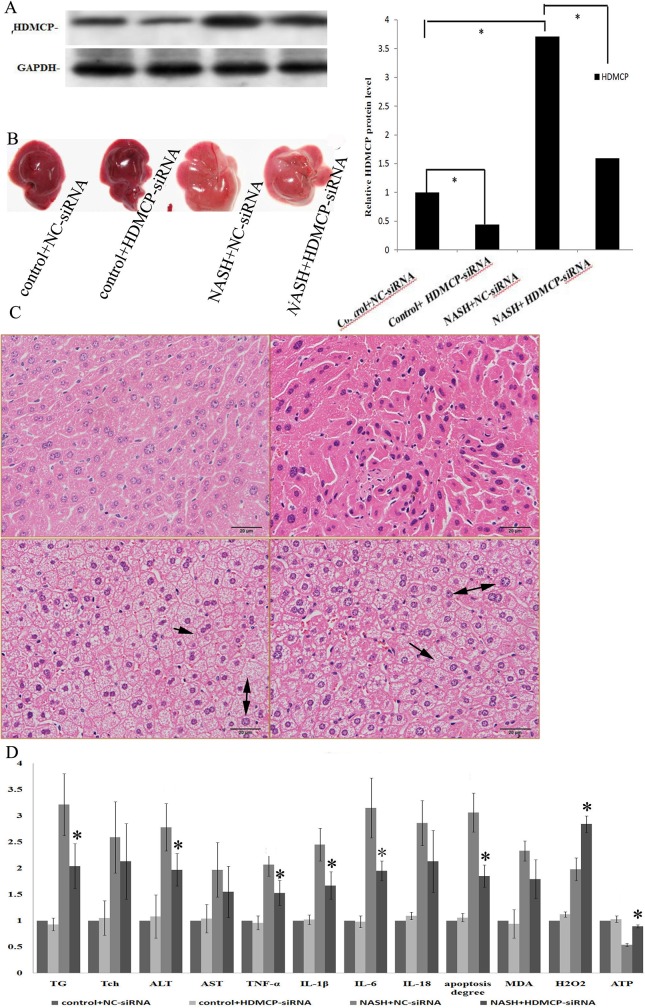
Change of pathology, steatosis and inflammation in NASH animalmodel after HDMCP interference. (A), western blot showed successful knockdown of HDMCP protein level by RNA interference. (B), MCD diet induced hepatic lipid deposition while HDMCP siRNA could antagonize this change. (C), H-E staining showed steatosis formation and inflammatory cell infiltration in MCD mice while HDMCP interference partially alleviated this trend. The left upper column represents control+NC-siRNA; the right upper column represents control+HDMCP-siRNA; the left lower column represents NASH+NC-siRNA; the right lower column represents NASH+HDMCP-siRNA. Single arrow indicates lipid droplet; double arrow indicates inflammatory cell infiltration. (D), Serum marker (steatosis, inflammation, oxidative stress related) change after HDMCP RNA interference. *, p<0.05. N = 8 for each group.

Using cholesterol modified HDMCP siRNAs, we successfully decreased HDMCP level in NASH+HDMCP siRNA group while NC-siRNA showed negative result, precluding the influence of non-specific siRNA in HDMCP expression (Fig 3A). Since HDMCP level was knocked down, the NASH degree in mice was significantly alleviated. Compared with NASH+NC-siRNA group, the size and yellowish degree of liver was apparently decreased in NASH+HDMCP-siRNA group, but not recovered to that of control group (Fig 3B). Further HE staining showed the decreased lipid droplet deposition and inflammation in NASH+HDMCP-siRNA group, compared with NASH+NC-siRNA group (Fig 3C). For statistical analysis in serum markers, we observed significant declination of serum TG, ALT, TNF-α, IL-1β, IL-6 levels and apoptosis degree in NASH+HDMCP-siRNA group, compared with NASH+NC- siRNA group. In addition, though Tch, AST, IL-18 and MDA levels were also decreased in NASH+HDMCP-siRNA group, it has not reached statistical significance when compared with NASH+NC-siRNA group (Fig 3D).

**Fig 4:**
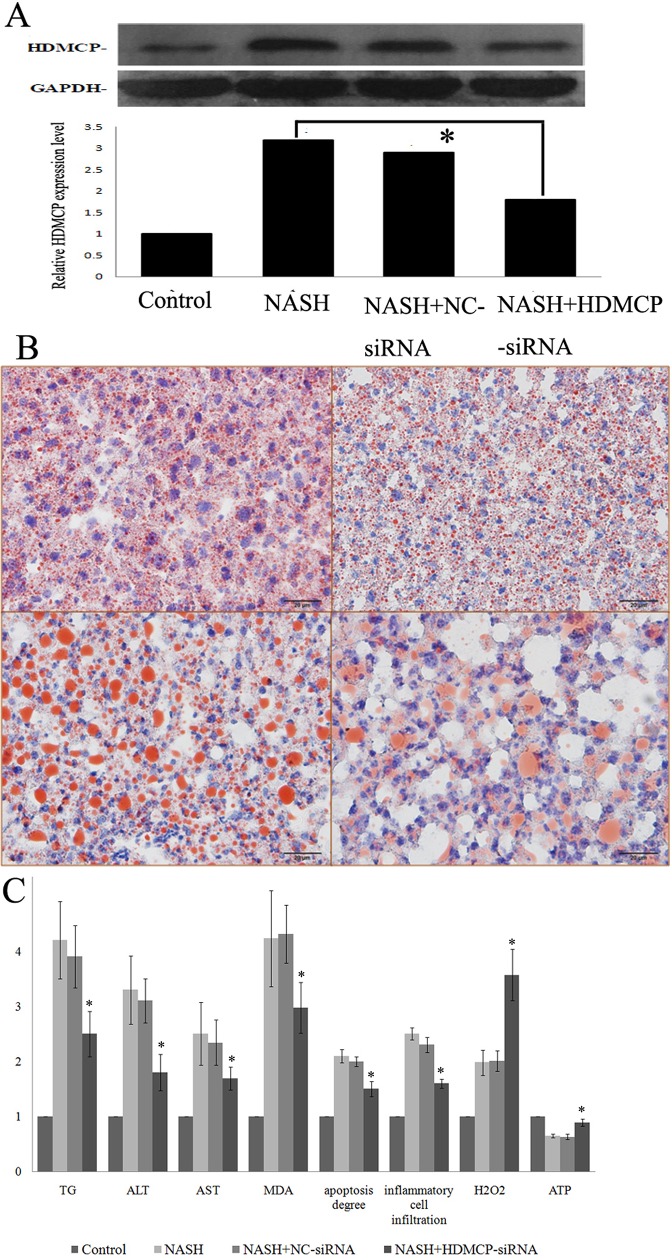
Change of steatosis and inflammation in HFFA-72h cultured L02 cell after HDMCP interference. (A), RNA interference mediated HDMCP protein level change through western-blot. (B), lipid droplet deposition (red droplet) in NASH cell model after oil-red staining. The left upper column represents control; the right upper column represents NASH; the left lower column represents NASH+NC-siRNA; the right lower column represents NASH+HDMCP-siRNA. (C), change of statistically significant markers (steatosis, inflammation, oxidative stress related) from HFFA-72h cultured L02 cell supernatant after HDMCP RNA interference. *, p<0.05. All cell experiments were carried out with three repetition.

We also successfully decreased HDMCP level in HFFA culutred L02 cell by RNA interference (Fig 4A). As shown in Fig 4B, the oil red stained droplet was significantly decreased in NASH+HDMCP-siRNA group, compared with NASH+NC -siRNA group. Moreover, the supernatant TG, ALT, AST, MDA levels, apoptosis degree and inflammatory cell infiltration degree were all significantly decreased in NASH+HDMCP-siRNA group, compared with NASH+NC-siRNA group (Fig 4C). HDMCP had the function of ATP dissipation and mitochondrial membrane potential (MMP) declination that was accountable for H_2_O_2_ formation. In this study, we observed the significantly increased ATP and H_2_O_2_ levels in NASH+HDMCP-siRNA groupsin both NASH mice (Fig 3D) and cell (Fig 4C) models, when compared with NASH+NC-siRNA groups.

### MiRNA-146 declination in NASH and its ability in HDMCP regulation

**Fig 5:**
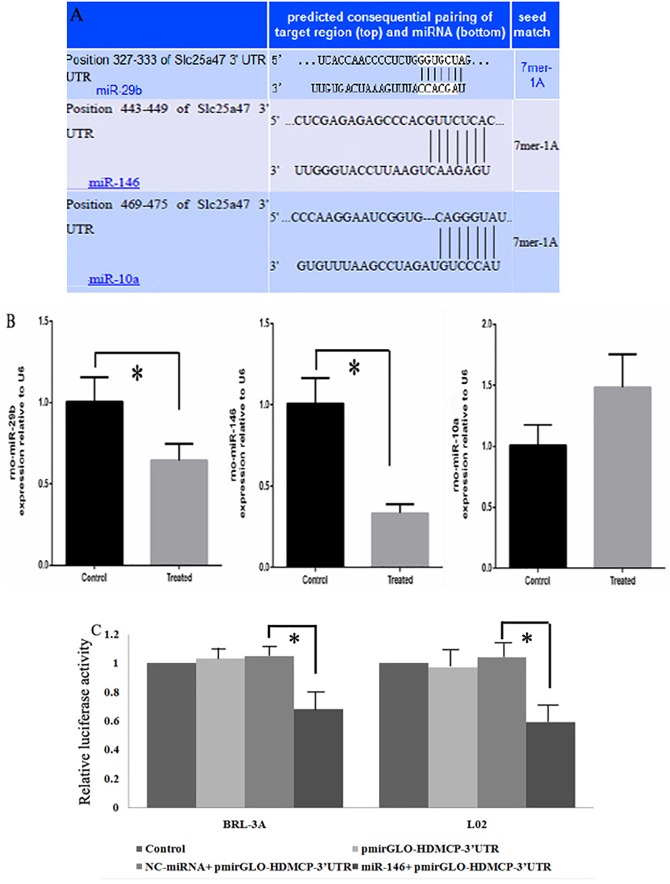
Regulation of miR-146 on HDMCP expression (A), prediction of miRNA binding site on the 3’ UTR of HDMCP. (B), relative change of miR-29b, miR-146 and miR-10a in fatty acid induced NASH cell model. (C), regulation of miR-146 on HDMCP in BRL-3A and L02 cells as shown by significantly decreased luciferase activity in miR-146+pmirGLO-HDMCP-3’UTR group. *, p<0.05.

Since our study showed increased HDMCP expression in both NASH cell and mice models and its potential ability in regulating NASH progression, its upstream miRNA might be decreased for its suppression ability in downstream protein regulation. Therefore, it is valuable to explore the potential miRNA-HDMCP-downstream effector pathway. We previously reported a panel of significantly decreased miRNAs in NASH rat model [20], where miR-146, miR-29b and miR-10a were predicted to regulate HDMCP through bioinformatics method (Fig 5A). For further verification, we first tested their levels in NASH cell model (BRL-3A hepatocyte cultured with HFFA for 72h) with qRT-PCR. As shown in Fig 5B, both miR-29b and miR-146 levels were significantly lower in HFFA treated group while miR-10a level was decreased but not reached statistical significance.

Since miR-146 had the highest degree of declination, we further chose it to test its ability in binding with HDMCP by dual luciferase detection method. In this step, we found that in both BLA-3A and L02 cells, the relative luciferase activity was significantly decreased in miR-146+pmirGLO-HDMCP-3’UTR group, compared with negative control miRNA+pmirGLO-HDMCP-3’UTR group (Fig 5C). Besides, there were no significant changes among control group (blank cell), pmirGLO-HDMCP-3’UTR group and negative control miRNA+pmirGLO-HDMCP- 3’UTR group. These results indicated the capacity of miR-146 in binding with HDMCP and called for further study in the effect of miR-146 in NASH pathogenesis and progress.

## Discussion

Currently, the pathogenesis of NASH is unclear, where mitochondrion has been considered to play a pivotal role and its change may be an adaptive response to the status of energy surplus in NASH[21]. The increased hepatic mitochondrial electron transport activity of obese mice[22] and decreased ATP level in NASH mice were reported [12], but the level and activity of ATP-synthase were unchanged. Previous studies identified the roles of UCPs in proton leak and ATP dissipation but their effect in NAFLD is still unclear [23]. Therefore, it is meaningful to explore the effect of UCP in NASH.

In this study, increased HDMCP level was identified in successfully established NASH mice model and HFFA cultured L02 cells (Table 1, Figs 1 and 2). Currently, the animal model of NASH is well developed[24]. However, though cell model of simple steatosis in NAFLD was reported through incubation with HFFA[25], the establishment of NASH cell model was still in blank. This may be due to the Lack of systematic evaluation including changes in cell apoptosis, inflammatory cell migration and oxidative stress. Based on our experience in setting up steatotic L02 and HepG2 cells[20], we further prolonged HFFA culture time to 72h and successfully observed classical changes that mimics NASH. Among them, Oil red staining and serum TG and Chol test were used to assess lipid accumulation. Besides, ALT and AST, mainly existing in cytoplasm and mitochondrion of hepatocyte, were used toevaluate hepatocyte injury. MDA is produced from lipid peroxidation and is biologically toxic for its ability of attacking and denaturing proteins, indirectly reflecting the degree of cellular free radical attack.

To increase the credibility of the NASH phenotype of HFFA cultured L02 cell model, assessment of cell apoptosis and inflammatory cell migration was carried out. Through flow cytometry, we found increased apoptosis level, another character of NASH, in HFFA cultured L02 cell. Furthermore, we found increased HL-60 cell migration in HFFA-72h cell groupby transwell method. The degree of HL-60 cell migration could well represent the ability of neutrophil infiltration and indirectly showed NASH severity. Neutrophil gradually migrated from hepatic vasculature to hepatocyte through different chemotactic factors such as TNF-α, adhesion molecule and endothelin[26].

Since Cheung O et al reported the association between NASH and altered hepatic miRNA [27], research on miRNA in NASH has been mushroomed, including its role as biomarker in diagnosis and player in disease progression [28]. Decreased miR-144 and miR-451 were found in NASH, where the former elicits and the latter inhibits proinflammatory cytokine production by respectively targeting TLR-2 [29]and AMPK/AKT pathway [30]. In contrast, increased miR-199a-5p was found in NASH by inhibiting nuclear receptor corepressor 1 translation[31]. Based on our microarray data of NASH rat model [20] and bioinformatics prediction (Fig 5A), we selected miR-146, miR-29b and miR-10ato detect their levels in NASH model. MiR-146 was finally chosen for its utmost fold change and the regulation of miR-146 on HDMCP was confirmed by dual luciferase detection. MiR-146 was identified to be associated with innate immune response [32] and breast cancer metastasis[33]. Therefore, it would be meaningful to study the effect of miR-146 in fibrosis and cirrhosis stages of NAFLD. Besides, since previous study reported the regulation of FOXP3 on miR-146/ NF-kB negative feedback loop[34], it is worthy studying such pathway in NASH. Finally, since ATP and H_2_O_2_levels have influenced lipid metabolism and inflammation process, it is also necessary to detect change of important genes such as SREBP, PPAR and leptin in NASH and their association with HDMCP.

For the uncoupling activity of HDMCP and its increased level in NASH, it is reasonable to test its therapeutic effect on NASH. In this study, we found alleviation of NASH in both in vivo and in vitro levels after RNA interference (Figs 3 and 4), supporting the potential therapeutic effect of antagonizing HDMCP in NASH. Since HDMCP is capable of uncoupling oxidative phosphorylation, we tested the downstream ATP and H_2_O_2_ change. As expected, ATP increment was paralleling with NASH alleviation (Fig 3D). However, in contrast with our previous result in simple steatosis [14], H_2_O_2_ level was increased in NASH models (Figs 3 and 4), indicating the existence of other factors that promote oxidative stress. Further increased H_2_O_2_ level in NASH alleviation by HDMCP downregulation demonstrated that increased HDMCP in NASH may be a protective factor by partially lowering H_2_O_2_ level. The potential mechanism may rely on the MMP associated oxidative stress that could be decreased by HDMCP upregulation [13]. Therefore, HDMCP in NASH progression may act as a seesaw. On one hand, the upregulated HDMCP induced ATP depletion may influence downstream genes in lipid metabolism and inflammation process as well as make the liver much more fragile to foreign blow; on the other hand, the ensuing MMP downregulation caused by HDMCP uncoupling may decrease the H_2_O_2_ production and act as a protective factor. Nevertheless, as we found a general NASH alleviation by HDMCP downregulation, such seesaw effect of HDMCP may slide toward the harmful effect of ATP depletion and targeting HDMCP as a whole controller might be effective in future NASH therapy.

There are several limitations of this study. Firstly, though MCD diet induced mice model has been widely used in NASH study, it is still not completely transferable to humans and the expression and effect of HDMCP in human NASH needs further study. Secondly, only miR-146 was selected in this study for its utmost fold change. However, previous study showed the capacity of MiR-29b in inhibiting collagen maturation in hepatic stellate cell[35], indicating its potential involvement in hepatic fibrosis. Therefore, its role in NASH and association with HDMCP need further investigation. Thirdly, since HL-60 cells do not share similarities with functional immune cells in human NAFLD, whether its neutrophil infiltration activity could represent the true process in NASH is still controversial. Besides, high dosage of HFFA could be toxic for hepatocytes and is not representative of in vivo situation. Therefore, though the HFFA-72h cultured L02 cell showed aspects of lipid deposition and inflammation activation, whether it could fully mimic all characters of NASH still needs cautious consideration. Fourthly, the HDMCP-3’UTR could not fully represent HDMCP, which impaired the deduction of regulator role of miR-146 on HDMCP. Finally, primary hepatocytes need to be studied in parallel to confirm that data generated by L02 cell line. In summary, we reported a miR-146-HDMCP-downstream effector pathway in NASH, which may provide novel mechanism and treatment option for NASH.

## Supporting information

S1 File Fig A in S1 File, **Graphics of plasmids and miRNA sequences for RNA interference** Fig B in S1 File, **Plasmid selection for in-vitro and in-vivo study.**
(PDF)
