# Supplementary material for: Effect of miR-146 targeted HDMCP up-regulation in the pathogenesis of nonalcoholic steatohepatitis
Source: PLoS One. 2017 Mar 27;12(3):e0174218. doi: 10.1371/journal.pone.0174218 (PMC5367781; doi:10.1371/journal.pone.0174218)
Supplement: S1 File — Fig A in S1 File, Graphics of plasmids and miRNA sequences for RNA interference Fig B in S1 File, Plasmid selection for in-vitro and in-vivo study. (PDF) [file pone.0174218.s001.pdf]

Title: Effect of miR-146 targeted HDMCP up-regulation in the pathogenesis of nonalcoholic steatohepatitis

[1]Xi Jin*, [2]Juan-wen Zhang#, [1]Yi-peng Chen, [1]Zun Xiang, [1]Jie-xia Ding, [1]You-ming Li

[1], Department of Gastroenterology, The First Affiliated Hospital, College of Medicine, Zhejiang University

[2],Department of Clinical Laboratory, The First Affiliated Hospital, College of Medicine, Zhejiang University

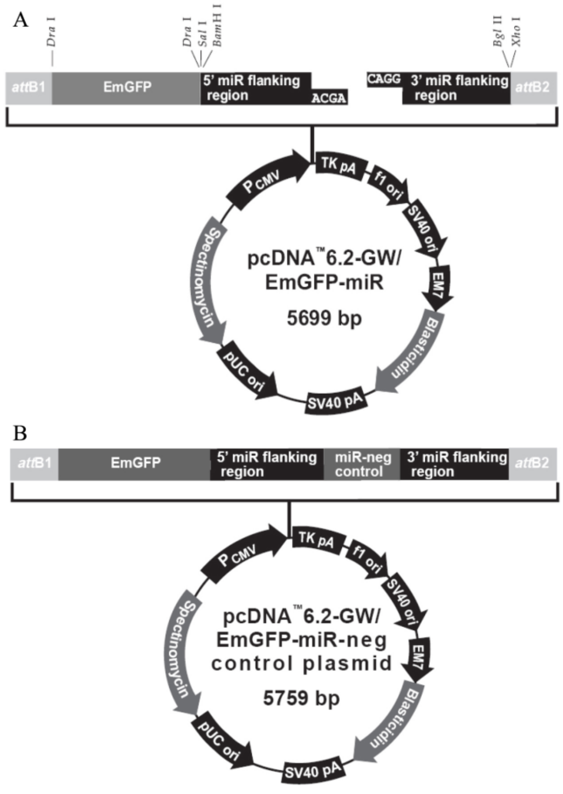

C

SLC25A47-F1-miR	5'- TGCTGAGTGCCAGATGCCTGTGTACTGTTTTGGCCACTGACTGACAGTACACACATCTGGCACT -3'
SLC25A47-R1-miR	5'- CCTGAGTGCCAGATGTGTGTACTGTCAGTCAGTGGCCAAAACAGTACACAGGCATCTGGCACTC -3'
SLC25A47-F2-miR	5'- TGCTGAGACACGGAAGATACCAGGGAGTTTTGGCCACTGACTGACTCCCTGGTCTTCCGTGTCT -3'
SLC25A47-R2-miR	5'- CCTGAGACACGGAAGACCAGGGAGTCAGTCAGTGGCCAAAACTCCCTGGTATCTTCCGTGTCTC -3'
SLC25A47-F3-miR	5'- TGCTGAACGCTGGTCACCATACAGTGGTTTTGGCCACTGACTGACCACTGTATTGACCAGCGTT -3'
SLC25A47-R3-miR	5'- CCTGAACGCTGGTCAATACAGTGGTCAGTCAGTGGCCAAAACCACTGTATGGTGACCAGCGTTC -3'
SLC25A47-F4-miR	5'- TGCTGAAGACCACCATGTTGACAGGGGTTTTGGCCACTGACTGACCCCTGTCAATGGTGGTCTT -3'
SLC25A47-R4-miR	5'- CCTGAAGACCACCATTGACAGGGGTCAGTCAGTGGCCAAAACCCCTGTCAACATGGTGGTCTTC -3'

D

ATGGATTTTGTCGCTGGAGCCATCGGAGGCGTCTGCGGTGTTGCTGTGGGCTACCCCCTGGACACGG
TGAAGGTCAGGATCCAGACGGAGCCAAAGTACACAGGCATCTGGCACTGCGTCCGGGATACGTATC
ACCGAGAGCGCGTGTGGGGCTTCTACCGGGGCCTCTCGCTGCCCGTGTGCACGGTGTCCCTGGTATCT
TCCGTGTCTTTTGGCACCTACCGCCACTGCCTGGCGCACATCTGCCGGCTCCGGTACGGCAACCCTGA
CGCCAAGCCCACCAAGGCCGACATCACGCTCTCGGGATGCGCCTCCGGCCTCGTCCGCGTGTTCCTG
ACGTCGCCCACTGAGGTGGCCAAAGTCCGCTTGCAGACGCAGACACAGGCGCAGAAGCAGCAGCGG
CGGCTTTCGGCCTCGGGGCCGTTGGCTGTGCCCCCCATGTGTCCTGTGCCCCCAGCCTGCCCAGAGCC
CAAGTACCGCGGGCCACTGCACTGCCTGGCCACGGTAGCCCGTGAGGAGGGGCTGTGCGGCCTCTAC
AAGGGCAGCTCGGCCCTGGTCTTACGGGACGGCCACTCCTTTGCCACCTACTTCCTTTCCTACGCGGT
CCTCTGCGAGTGGCTCAGCCCCGCTGGCCACAGCCGGCCAGATGTCCCGGGCGTGCTGGTGGCCGGG
GGCTGTGCAGGAGTCCTGGCCTGGGCTGTGGCCACCCCCATGGACGTGATCAAGTCGAGACTGCAGG
CAGACGGGCAGGGCCAGAGGCGCTACCGGGGTCTCCTGCACTGTATGGTGACCAGCGTTCGAGAGG
AGGGACCCCGGGTCCTTTTCAAGGGGCTGGTACTCAATTGCTGCCGCGCCTTCCCTGTCAACATGGTG
GTCTTCGTCGCCTATGAGGCAGTGCTGAGGCTCGCCCGGGGTCTGCTCACATAG

Supplementary Figure 1, Graphics of plasmids and miRNA sequences for RNA interference. (A), schematic representation of pcDNA™6.2-GW/EmGFP-miR expression plasmid; (B), schematic representation of pcDNA™6.2-GW/ EmGFP-miR-neg control expression plasmid; (C), four sequences of inserted miRNAs to antagonize HDMCP expression, where the underlined third sequence was finally selected; (D), four binding sizes for miRNA sequence 1-4 was sequentially marked as yellow, red, blue and green in HDMCP.

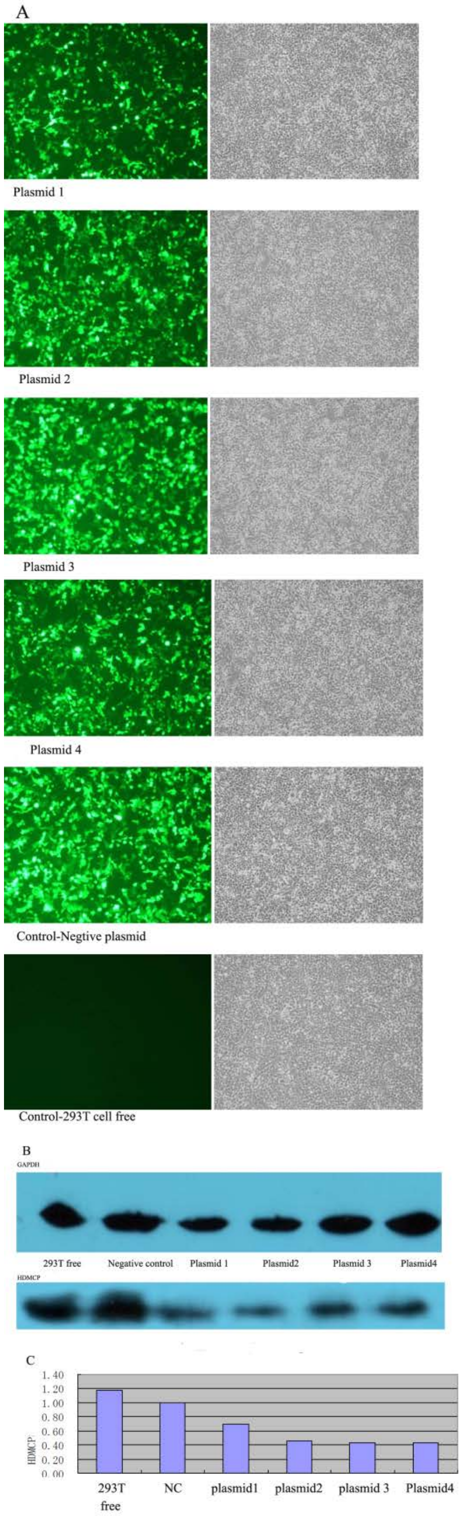

Supplementary Figure 2, Plasmid selection for in-vitro and in-vivo study. (A), fluorescence photo of 293T cell transfected with different reagents; (B), HDMCP expression after RNA interference shown by western blot.
